# Tobacco smoking is associated with decreased semen quality

**DOI:** 10.1186/s12978-016-0207-z

**Published:** 2016-08-05

**Authors:** H. Asare-Anane, S. B. Bannison, Emmanuel K. Ofori, R. O. Ateko, A. T. Bawah, S. D. Amanquah, S. Y. Oppong, B. B. N. Gandau, J. B. Ziem

**Affiliations:** 1 Department of Chemical Pathology, School of Biomedical and Allied Health Sciences (S.B.A.H.S), University of Ghana, Korle-Bu Accra, Ghana; 2 Chemical Pathology Unit, Tamale Teaching Hospital, Tamale, Ghana; 3 Department of Medical Laboratory, U.H.A.S, Ho, Ghana; 4 Department of Chemical Pathology, School of Biomedical and Allied Health Sciences (S.B.A.H.S), University of Ghana, Accra, Ghana; 5 Department of Obstetrics and Gynaecology, S.M.H.S, University for Development Studies, Tamale, Ghana; 6 Department of Medical Laboratory Science, S.M.H.S, University for Development Studies, Tamale, Ghana

**Keywords:** Cigarette smoking, Semen quality, Sperm morphology, Hydrocarbon

## Abstract

**Background:**

Tobacco smoking is a public health issue and has been implicated in adverse reproductive outcomes including semen quality. Available data however provides conflicting findings. The objective of this study was to evaluate the effect of tobacco smoking on semen quality among men in Ghana.

**Methods:**

In this study, a total of 140 subjects were recruited, comprising 95 smokers and 45 non-smokers. Smokers were further categorized into mild, moderate and heavy smokers. Semen parameters such as sperm concentration, motility, viability and normal morphology were measured according to the World Health Organisation criteria.

**Results:**

The study showed that smokers had significantly lower semen volume, sperm concentration, sperm motility, total sperm count, sperm morphology, free testosterone and follicle stimulating hormone (*p* <0.05 respectively), compared with non-smokers. Smokers were at a higher risk of developing oligospermia, asthenozoospermia and teratozoospermia (OR = 3.1, 4.2 and, 4.7; *p* <0.05) than non-smokers.

**Conclusion:**

Results demonstrated a decline in semen quality in a dose dependent tobacco smoking manner.

**Electronic supplementary material:**

The online version of this article (doi:10.1186/s12978-016-0207-z) contains supplementary material, which is available to authorized users.

## Background

Lifestyle factors such as smoking, alcohol consumption, drug abuse as well as increased levels of environmental pollutants have been suggested as factors responsible for decline in semen quality [1–6]. Cigarette smoking is a major public health problem [7]. The highest prevalence of smoking is observed in young adult males in their reproductive period [8]. Cigarette smoking may be associated with sub-fertility in males, which may be attributed to decreased sperm concentration, lower sperm motility and a reduced percentage of morphologically normal sperms [9–11]. The Ghana Statistical Service (GSS), in its demographic and health survey report stated that the three Northern regions topped Ghana in tobacco usage [12]. The Northern Region recorded 17.7 % of men engaged in smoking, the Upper West Region 15.3 % and the Upper East Region 11.4 % [12]. With developing countries like Ghana being at high risk of epidemic increases in tobacco smoking in addition to the lack of data on the effect of tobacco smoking on semen quality in Ghana, investigations into this area of research is necessary. The objective of this study was to evaluate the effect of tobacco smoking on semen quality. We hypothesised that tobacco smoking was not associated with decreased semen quality.

## Methods

### Study site and design, participants, inclusion and exclusion criteria

The study was carried out at the Tamale Teaching Hospital located in the Northern Region of Ghana. This was a cross-sectional study with matched controls. The University of Ghana Medical School, Ethical and Protocol review committee reviewed and approved the study. A written informed consent was obtained from all subjects before their participation. The study was conducted from January 2010 to April 2011. A total of 801 men in communities within the Tamale metropolis, were spoken to and 433 men agreed to participant in the study. Two hundred ninety-three men were further excluded from the study based on the following: Subjects visiting fertility clinic, with varicocele, history of testes injury, occupational exposure and use of pesticides, subjects with a history of chronic urinary tract infection, subjects with disorders such as diabetes mellitus, hypertension and coronary heart diseases. Finally, 140 men between the ages of 18 and 45 years were involved in the study. Participant’s habits and admittance to the use of known banned pesticides from our questionnaire primarily helped us in the selection process. Most of the participants were heads of households, with no formal education and were engaged in subsistence farming. Majority of the men had been married for at least 4 years and were with at least a child. The youngest children from these men were aged between 6 weeks to 4 years.

Subjects were classified into two main groups: non-smokers (control group) and smokers. Smokers were defined as subjects who have smoked tobacco (and are still smoking) continuously for at least 6 months. The smokers were further stratified into mild, moderate and heavy smokers depending on the number of sticks of cigarette smoked per day. Smokers who smoked less than five (<5) sticks of cigarette per day were classified as mild smokers, between 5 and 10 sticks of cigarette per day as moderate smokers and more than ten (>10) sticks per day as heavy smokers. Detailed medical history and examination was conducted by an urologist to eliminate men with conditions that could affect fertility. Demographic data, average number of cigarette sticks smoked per day, the duration of smoking and ages of participants were recorded using a questionnaire.

### Minimum sample size

Using 10 % proportion of Ghanaian men who smoked actively, an acceptable error of 15 %, a power of 80 % and a significance level of 95 %, we estimated that a sample size of 15 in each sub group (mild, moderate, heavy smokers and non-smokers) would be adequate for the study.

### Collection of semen samples

The semen samples were obtained by masturbation, after subjects had abstained from sex or ejaculation for a period of at least 3 days. The ejaculate was collected into a clean, dry, sterile, leak proof container with a wide mouth to prevent spillage. Samples with partial spillage were rejected. Semen samples of such subjects were taken again after 3–6 days’ abstinence. Semen samples were allowed to liquefy in an incubator at 37 °C and analysed according to World Health Organizations stipulated guidelines [13].

### Blood sampling and analysis

Due to diurnal variations in hormonal concentrations, venous blood samples were collected between 6 am and 8 am after a 10–14 h fast. Of the 5 ml of blood drawn, 4 ml was transferred into serum separator tubes and processed for reproductive hormonal analysis. The resulting sera were stored at −20 °C until required for use. The remaining 1 ml was transferred into sodium fluoride containing tube for the estimation of glucose. Serum total testosterone (TT), sex hormone binding globulin (SHBG) estradiol (E_2_), luteinizing hormone (LH), and follicle-stimulating hormone (FSH) were determined by enzyme-linked immunosorbent assay (Kamiya Biomedical Company, USA.). A calibration curve was used to determine the analyte concentration from the strength of signal produced in the immunoassay. A specific secondary antibody was used to eliminate non-specific binding to substrates.

## Semen analysis

### Measurement of semen volume

Liquefied semen sample volume was measured by aspirating the entire sample into a graduated syringe.

### Measurement of pH

Well mixed liquefied semen sample was dropped onto a narrow range pH paper (6.4–8.0) and the result read and recorded after 60 s.

### Assessment of sperm motility

For the assessment of sperm motility, the sample was well mixed and 10 μl transferred onto a clean glass slide (which had been kept at 37 °C in an incubator). The preparation was covered with a 22 × 22 mm coverslip, placed on the stage of a microscope and immediately examined at a magnification of ×40. The motility assessment was repeated on a second aliquot of semen and an average value was taken.

### Assessment of sperm concentration

Semen sample was thoroughly mixed for 5 min in a rotation device and 1:20 dilution was made using sodium bicarbonate formalin solution. Twenty (20)μl of the diluted specimen was transferred onto an improved Neubauer ruled haemocytometer. The sample was allowed to stand for 10 min in a humid chamber for the cells to settle. Four chambers of the haemocytometer were counted, and the average was used in the analysis [13].

### Assessment of sperm morphology

#### Stained preparation

Twenty (20)μl of well mixed liquefied semen sample was placed on a clean slide (two per semen sample). A thin smear was prepared and allowed to air-dry. The smear was fixed in 95 % v/v ethanol for 5 min and allowed to air-dry. The slide was washed in sodium bicarbonate formalin solution to remove any mucus which may be present, then rinsed in water and covered in diluted (1 in 20) carbol fuchsin for 3 min. The stain was washed off in water. Diluted (1 in 20) Loeffler’s methylene blue was used to counter stain the smear for 2 min. The counter stain was washed in water and the smear was allowed to air-dry. Using the ×100 objective, 100 sperms were observed and the number of sperms showing normal and abnormal morphology was counted and recorded.

### Statistical analysis

Comparisons between mild, moderate and heavy smokers were performed using one-way analysis of variance (ANOVA) and unpaired *t*-test was used for comparison between smokers and non-smokers. All hypothesis testing were two-tailed with a significance level of 0.05. All statistical analysis was performed with the GraphPad version 5.0 (GraphPad Software, San Diego, CA). A sample size of 15 in each sub group (mild, moderate, heavy smokers and non-smokers) was enough for between group comparisons and gave a power of 0.8.

## Results

### General characteristics of the study population

The clinical and biochemical parameters of the study population are shown in Table 1. A total of 140 subjects were recruited of which, 45 (32.1 %) were non-smokers while 95 (67.9 %) were smokers. Thirty (30) each of the smokers (31.6 %) were mild and moderate smokers respectively, while 35 of the smokers (36.8 %) were heavy smokers (Table 1). There was no significant difference (*p* = 0.2437) in the mean ages of smokers and non-smokers (Table 1). The mean semen volume, percentage motility, normal sperm morphology, total sperm count, follicle stimulating hormone (FSH), free and total testosterone (TT) of non-smokers were significantly higher (*p* = 0.028, *p* = 0.034, *p* = 0.038, *p* = 0.041, *p* = 0.007, *p* = 0.021, *p* = 0.039) when compared with smokers. The mean sluggishly motile sperms and percentage immobile sperms were significantly lower in non-smokers than smokers respectively (*p* = 0.043; *P* = 0.012). Sperm viability, number of epithelial cells (ECs), pus cells (PCs), red blood cells (RBCs), luteinizing hormone (LH), Estradiol (E_2_) and prolactin (PROL) in the semen of non-smokers and smokers did not show any significant difference (*P* >0.05).

**Table 1:** Clinical and biochemical parameters of smokers stratified by the number of sticks smoked per day

Parameters	Non-smokers (*N* = 45)	Mild (*N* = 30)	Moderate (*N* = 30)	Heavy (*N* = 35)	*p*-value
Age (years)	35.0 ± 5.4	35.0 ± 7.4	37.3 ± 7.4	37.6 ± 9.3	0.847
Volume (ml)	3.37 ± 1.0	2.7 ± 0.7^a^	2.2 ± 0.4^b^	2.6 ± 0.3^c^	0.028*
pH	7.70 ± 0.2	7.70 ± 0.3	7.60 ± 0.8	6.80 ± 1.5	0.736
Immobile (%)	7.64 ± 2.3	35.1 ± 9.8^a^	36.2 ± 8.7^b^	34.5 ± 10.1^c^	0.012*
Active (%)	53.76 ± 12.4	48.3 ± 11.7	41.4 ± 13.7^b^	24.9 ± 9.5^c, d^	0.034*
Sluggish (%)	10.36 ± 4.5	16.6 ± 6.7^a^	16.0 ± 5.5^b^	19.6 ± 7.6^c^	0.043*
Viability (%)	65.0 ± 14.8	65.1 ± 13.1	60.3 ± 15.7	46.8 ± 17.2^c, d^	0.284
Morphology (%)	48.40 ± 12.1	43.8 ± 9.5	38.8 ± 10.2^b^	26.1 ± 10.4^c, d^	0.038*
Count (sperm/ml)	93.17 ± 22.1	73.6 ± 21.6^a^	64.3 ± 19.8^b^	24.5 ± 12.1^c^	0.041*
PC(cells/HPF)	5.7 ± 1.2	6.9 ± 2.1^a^	6.2 ± 1.8	6.2 ± 2.7	0.436
EC (cells/HPF)	2.0 ± 0.9	2.4 ± 1.1	2.1 ± 0.8	2.4 ± 0.7	0.865
RBC (cells/HPC)	0.08 ± 0.0	0.1 ± 0.0	0.1 ± 0.0	0.1 ± 0.0	0.973
FSH (IU/L)	8.56 ± 2.8	6.90 ± 2.6^a^	6.80 ± 2.7^b^	5.80 ± 2.1^c^	0.007*
LH (IU/L)	6.24 ± 1.9	6.10 ± 2.4	6.00 ± 1.9	5.60 ± 1.2^c^	0.439
TT (nmol/L)	18.8 ± 4.6	14.9 ± 3.2	14.5 ± 5.7	13.8 ± 5.6	0.039*
FT (nmol/L)	0.36 ± 0.1	0.27 ± 0.1^a^	0.26 ± 0.1^b^	0.24 ± 0.1^c^	0.021*
Bio T (nmol/L)	8.43 ± 2.9	6.21 ± 2.2^a^	5.82 ± 2.0^b^	5.50 ± 1.4^c^	0.018*
SHBG (nmol/L)	39.6 ± 7.3	41.8 ± 11.6	42.4 ± 5.0	43.2 ± 9.3	0.389
E_2_ (pg/ml)	23.0 ± 9.2	24.5 ± 9.5	25.3 ± 10.1	25.9 ± 10.8	0.691
PROL (ng/ml)	15.0 ± 3.6	15.1 ± 4.4	16.8 ± 2.8	17.3 ± 3.1	0.352

The results for the clinical and biochemical parameters of the study population

Values are given as mean ± standard deviation. *mean difference is significant (*p* <0.05). *PC* pus cells, *EC* epithelial cells, *RBC* red blood cells, *FSH* follicle stimulation hormone, *LH* luteinizing hormone, *TT* total testosterone, *FT* free testosterone, *Bio T* bioavailable testosterone, *SHBG* sex hormone binding globulin, *E2* estradiol and PROL is prolactin. ^a^ *p* <0.05for corresponding values of mild smokers with controls; ^b^ *p* <0.05 for comparison of moderate smokers with controls; ^c^ *p* <0.05 for corresponding values of heavy smokers with non-smokers; ^d^ *p* <0.05 for corresponding values of heavy smokers with mild smokers

### The effect of number of sticks smoked per day on semen parameters

This study investigated the effect of number of cigarette sticks smoked per day on semen parameters (Table 2). Beta (β) is the slope of the line of best fit and shows the magnitude of effect of a unit change in the number of sticks smoked per day on semen parameters. The strength of linear association between numbers of sticks smoked per day on semen parameters is denoted by r. The results showed that the number of cigarette sticks smoked per day had no significant effect on semen volume, sluggishness, PC, EC and RBC (*p* = 0.667, 0.358, 0.236, 0.650, 0.491 respectively), but significantly reduced semen pH, sperm motility, viability, morphology, count and total sperm count (*p* <0.001, *p* = 0.004, 0.002, 0.001, 0.019, 0.037 respectively) (Table 2).

**Table 2:** Effect of cigarette sticks smoked per day on semen parameters

Semen parameters	Number of sticks		
	β	r^2^	*p*-value
Volume (ml)	−0.01	0.00	0.667
pH	−0.13	0.42	0.001
Active (%)	−1.31	0.15	0.004
Sluggish	0.22	0.01	0.358
Immobile	−0.60	0.05	0.093
Morphology (%)	−1.10	0.19	0.001
Viability (%)	−1.44	0.17	0.002
Count (sperm/ml)	−2.70	0.10	0.019
Total count (# of sperm)	−7.43	0.07	0.037
PC (cells/HPF)	0.08	0.02	0.236
EC (cells/HPF)	0.01	0.00	0.650
RBC (cells/HPF)	−0.01	0.01	0.491

Shows linear regression of the effect of number of cigarette sticks smoked per day and the duration of the smoking on semen parameters. *β* slope of the line of best fit, *r* ^*2*^ coefficient of determination, *PC* pus cells, *EC* epithelial cells, *RBC* red blood cells, *#* number

### Semen abnormalities among smokers and non-smokers

This study compared the risk of developing semen abnormality among non-smokers and smokers (Table 3). Smokers significantly had higher: oligospermia, asthenospermia and teratozoospermia (*p* = 0.047, 0.001 and 0.0003 respectively); reduced viability and total sperm count, (*p* = 0.0041 and 0.0076 respectively) compared with non-smokers and were at higher risk of developing oligospermia, asthenospermia and teratozoospermia (3.1, 4.2 and, 4.7 times respectively) compared with non-smokers. The percentage of smokers with reduced sluggish sperm was significantly lower compared with non-smokers (*p* = 0.0323), with smokers being 0.4 times at risk of having sluggish sperms compared to non-smokers.

**Table 3:** Risk of developing semen abnormalities among smokers and non-smokers

Semen abnormality	Smokers (%)	Non-smokers (%)	Odds ratio	*p*-value
Reduced semen volume	31.5	15.8	2.1	0.1180
Oligospermia	27.8	11.1	3.1	0.0047
Asthenozoospermia	57.4	24.4	4.2	0.0010
Teratozoospermia	76.0	39.8	4.7	0.0003
Reduced viability	87.0	62.2	4.1	0.0041
Reduced sperm count	37	13	3.8	0.0076
Reduced sluggish	77.8	57.4	0.4	0.0323

The risk associated with semen abnormality in the study population. *P* <0.05 is significant

## Discussion

Findings from this study underscore the fact that tobacco smoking has adverse reproductive outcome on semen quality. The relationship between smoking and levels of total testosterone (TT) and Sex hormone binding globulin (SHBG) were investigated in this study. Smoking in this study associated significantly with reduced TT levels. This finding corroborated with a prior study [14], but disagreed with another [15]. The mechanism mediating the effect of smoking on testosterone production is not fully understood, however, it could be due to the toxic effect of cigarette smoking on leydig cells, directly reducing testosterone biosynthesis [14]. Others have suggested that smoking increases testosterone levels by reduced conversion to estradiol [16]. This however was not observed in the present study. No significant difference was observed in SHBG levels between non-smokers and smokers, which agreed with earlier reports [17, 18] but disagreed with others [15, 19]. The results in this study showed that semen volume, percentage motility, morphology, concentration (count), and total count were all lower in smokers compared with non-smokers (Table 1). The observed reductions in semen volume in smokers compared with non-smokers have been reported by others [20–22]. Results from this study further showed that the reduction in semen volume in smokers was inversely proportional to the number of sticks smoked per day. This could be the result of the presence of nicotine in cigarette, which affects the functioning of accessory sex glands (seminal vesicle, prostate and urethral glands), that control semen volume through their secretions [23]. In this study, sperm motility was significantly lower in smokers compared with non-smokers. This finding was supported by previous studies [7, 10, 11]. This is the result of the mutagenic effects of aromatic hydrocarbons [24], and the toxic effects of nicotine [25] that can disrupt the testicular microcirculation, thereby reducing the number of red blood cells reaching the cells of the testes to supply oxygen for respiration. Carbon monoxide found in cigarette smoke is also known to reduce availability of oxygenated haemoglobin, leading to a reduced supply of oxygen to the sperm cell [26] which results in a decreased utilization of oxygen by the mitochondria of the sperm cells [27] thereby reducing the motility of the sperms, hence sluggish sperms. The sperm tail contains lots of mitochondria; therefore, ultra-structural damage in the tail region as a result of smoking can impair mitochondrial function.

On sperm morphology, the results of this study showed that smokers had significantly lower normal sperm morphology compared with non-smokers and the frequency of abnormal morphology increased as the number of tobacco sticks smoked per day increased; putting smokers at a higher risk of developing teratozoospermia. This study was in agreement with others [28, 29] who reported that cigarette smoking increases the percentage of morphologically altered spermatozoa. This is as a result of the presence of toxic chemicals, mutagenic and carcinogenic compounds found in cigarette smoke, which adversely affect sperm morphology [30].

Several studies have reported that the mutagenic components of cigarette smoke adversely affected rapidly dividing cells, including germ cells in the testis [23, 31]. MacKenzie and Angeline [32] demonstrated that polycyclic aromatic hydrocarbons and nicotine present in cigarette smoke can cause atrophy of seminiferous tubules, testis and reduce or block spermatogenesis. In this study, the significantly lower sperm concentration, total sperm concentration, in smokers compared with non-smokers were the results of mutagenic compounds present in cigarette. Total sperm count which is a function of semen volume and sperm count was also significantly lower in smokers than in non-smokers. The study further revealed that smokers were at higher risk of developing oligospermia compared with non-smokers and that the effect was dose-dependent. This corroborated with a prior study [33]. The presence of nicotine in cigarette impairs spermatogenesis that makes smokers to be at higher risk of developing reduced total sperm count as compared with non-smokers [14].

Semen abnormalities (oligospermia, asthenozoospermia and teratozoospermia) were present in this study. The results showed that semen abnormalities were more prevalent among heavy smokers compared to moderate and mild smokers. These abnormalities were dose-dependent and agreed with prior studies [34, 35]. At the time of conducting this study, there was difficulty in getting facility to estimate serum nicotine. Serum nicotine would have been a better measure of the dose effect of tobacco smoking than the number of sticks smoked per day (Additional file 1).

## Conclusion

This study showed that smoking significantly reduced semen volume, sperm viability, sperm motility, sperm morphology and concentration. Smoking reduced semen quality in a dose-dependent manner.

## Abbreviations

Ast, asthenozoospermia; Bio T, bioavailable testosterone; E_2_, estradiol; FSH, follicle stimulating hormone; FT, free testosterone; GSS, Ghana Statistical Service; LH, luteinizing hormone; PC, pus cells; PROL, prolactin; R.Tc, reduced total sperm count; R.Via, reduced viability; RBC, red blood cells; SHBG, sex hormone binding globulin; Ter, teratozoospermia; TT, total testosterone; WHO, world health organization

## Additional file

Additional file 1: Raw Data. (XLSX 34 kb)
